# The Role of Bioactives in Inflammation

**DOI:** 10.3390/cimb46030125

**Published:** 2024-02-29

**Authors:** Po-Chih Hsu, Guan-Ting Liu, Jiunn-Sheng Wu, Chan-Yen Kuo

**Affiliations:** 1 Department Dentistry, Taipei Tzu Chi Hospital, Buddhist Tzu Chi Medical Foundation, New Taipei City 23142, Taiwan; pino0906@gmail.com; 2 Institute of Oral Medicine and Materials, College of Medicine, Tzu Chi University, Hualien 970374, Taiwan; 3 Department of Research, Taipei Tzu Chi Hospital, Buddhist Tzu Chi Medical Foundation, New Taipei City 23142, Taiwan; mochabear19@gmail.com; 4 Division of Infectious Diseases, Taipei Tzu Chi Hospital, Buddhist Tzu Chi Medical Foundation, New Taipei City 23142, Taiwan; wujs.medoc@gmail.com

As a physiological defense mechanism, inflammation is a complex response to harmful stimuli. Nevertheless, chronic diseases may develop as a result of chronic inflammation if it is prolonged and excessive in nature [1]. Accumulating recent studies have suggested that natural bioactives play a significant role in inflammation and disease, improving our wellbeing and even improving our quality of life by reducing the risks associated with inflammatory diseases [2,3,4,5]. Inflammatory diseases can be effectively treated with different types of bioactives using different research models and methodologies. It is our pleasure to invite five articles in 2023 highlighting the current status of anti-inflammatory compounds, natural products, synthesized compounds, as well as novel mechanisms and targets in regard to stopping inflammation.

Park et al. demonstrated that the extract of *Sargassum honeri* along with fucoidan prevents hyperosmotic stress-induced apoptosis of corneal epithelium and lacrimal glands, thus preventing tears from hyposecretion and apoptosis. Patients suffering from dry eye disease may benefit from the use of *S. honeri* extract and fucoidan [6].

The antioxidant properties of orange flesh sweet potato flour (OFSP) flour extract, as well as its immunomodulatory potential on inflammatory murine macrophages, were investigated. This study specifically examined the effect of OFSP flour extract on mediators such as nitric oxide (NO) as well as pro-inflammatory cytokines such as Interleukin-6 (IL-6), Tumor Necrosis Factor alpha (TNF-alpha), Monocyte Chemoattractant Protein-1 (MCP-1), and Prostaglandin-E2 (PGE-2). The results demonstrated that the extracts possessed antioxidant and immunomodulatory bioactivities, with a concentration-dependent inhibition of cytokine production. Furthermore, the extract exhibited significant levels of fiber, mineral, beta-carotene, and polyphenols, supporting its potential therapeutic value. In summary, *Ipomoea batatas* flour could provide energy and beta-carotene provitamin A and be a food of interest that can help prevent the occurrence of metabolic diseases associated with an underlying state of low-grade inflammation [7].

A mortality rate of up to 40% is associated with acute kidney injury (AKI) caused by sepsis. Several mechanisms are involved in the pathogenesis of septic AKI, which lead to an increase in inflammation and renal dysfunction [8]. Inflammatory disorders can be treated with eupatilin (EUP), which is a natural flavone that exerts multiple biological activities. However, the effectiveness of EUP in treating septic AKI remains to be determined. Kim et al. examined the effects of EUP on lipopolysaccharide (LPS)-induced acute kidney injury in mice. The results indicated that EUP attenuated the effects of LPS on renal dysfunction, as shown by reductions in serum creatinine and blood urea nitrogen concentrations. Renal function was restored by EUP. As a result of LPS injection, the proximal tubules lost their brush borders, and tubular injury markers including neutrophil gelatinase-associated lipocalin (NGAL) and kidney injury molecule-1 (KIM-1) were upregulated. The LPS injection also led to tubular cell detachment, tubular dilatation, and tubular cell death. These phenomena were attenuated by EPU treatment. There was also a decrease in serum and renal cytokine levels in response to EUP, and macrophage infiltration was prevented upon EUP treatment. Despite this, EUP mitigated LPS-induced oxidative stress by downregulating NPDPH oxidase 4 and upregulating antioxidant enzymes. LPS-treated mice were also inhibited by EUP in p53-mediated apoptosis. Hence, EUP inhibits oxidative stress, inflammation, and apoptosis, thereby ameliorating LPS-induced AKI [9].

Sun et al. studied the effects of silibinin, derived from milk thistle *Silybum marianum*, on lipopolysaccharide (LPS)-induced morphological changes in mice macrophages. The results demonstrated that silibinin inhibited pseudopodia formation and the size increase that was induced by LPS, while unstimulated cells remained round. During LPS-stimulated macrophage phagocytosis, silibinin inhibited phagocytosis. Using an MAPK array, the authors also investigated the mechanism of action of the compound on kinases. Silibinin exhibited a more pronounced inhibition of ERK1/2 and related RSK1/2 compared to JNK and p38, which are the three MAPK family members that were tested. The expression of MKK6, AKT3, MSK2, p70S6K, and GSK-3β was downregulated in the presence of silibinin. Moreover, silibinin demonstrated significant inhibitory effects on macrophage phagocytosis and induced changes in macrophage morphology. The authors speculated that silibinin may serve as an anti-inflammatory agent, as it targets key inflammatory pathways involving ERK1/2 and related kinases. As supported by the findings of this study, silibinin possesses promising therapeutic properties in modulating macrophage function and controlling inflammation (contribution 1).

The *Chisocheton* plant is a member of the Meliaceae family and has traditionally been used to treat various diseases; however, there is insufficient scientific evidence to substantiate its medicinal use. These plants contain limonoids, which have been found to have a variety of biological effects, including anti-inflammatory properties. Nevertheless, constituents of *Chisocheton* plants have not been fully investigated for their anti-inflammatory effects or the underlying mechanisms of action. Using an ELISA assay, Hilmayanti et al. investigated the anti-inflammatory activities of 17 limonoid compounds derived from the *Chisocheton* plant. The group evaluated these compounds for their inhibitory effects on the production of pro-inflammatory cytokines, including TNF, IL-6, IL-1, and MCP-1, in THP-1 cells stimulated with LPS. Among the 17 tested compounds, Compounds 3, 5, 9, and 14–17 demonstrated significant inhibition of pro-inflammatory markers, with IC_50_ values less than 20 m and a high selectivity index (contribution 2).
